# Supplementary material for: Identification of photoperiod-induced specific miRNAs in the adrenal glands of Sunite sheep (Ovis aries)
Source: Front Vet Sci. 2022 Jul 22;9:888207. doi: 10.3389/fvets.2022.888207 (PMC9354845; doi:10.3389/fvets.2022.888207)
Supplement: Supplementary file 1 [file Data_Sheet_1.ZIP › Supplementary file/miRDeep2 file.zip!/miRDeep2 file/Novel_Structure/SPLP42A2/Novel_299.pdf]

Provisional ID	:	NC_019465.2_18894
Score total	:	1.2
Score for star read(s)	:	-1.3
Score for read counts	:	0
Score for mfe	:	0.9
Score for randfold	:	1.6
Score for cons. seed	:	
Total read count	:	23
Mature read count	:	23
Loop read count	:	0
Star read count	:	0

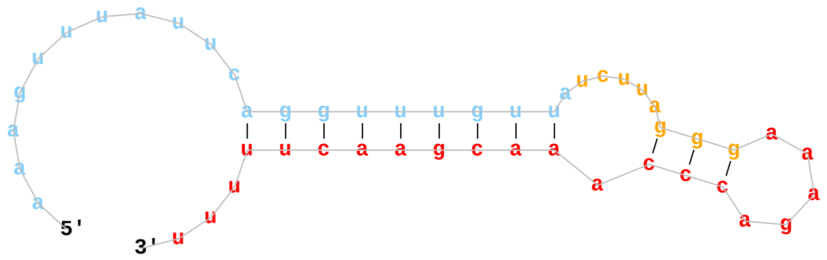

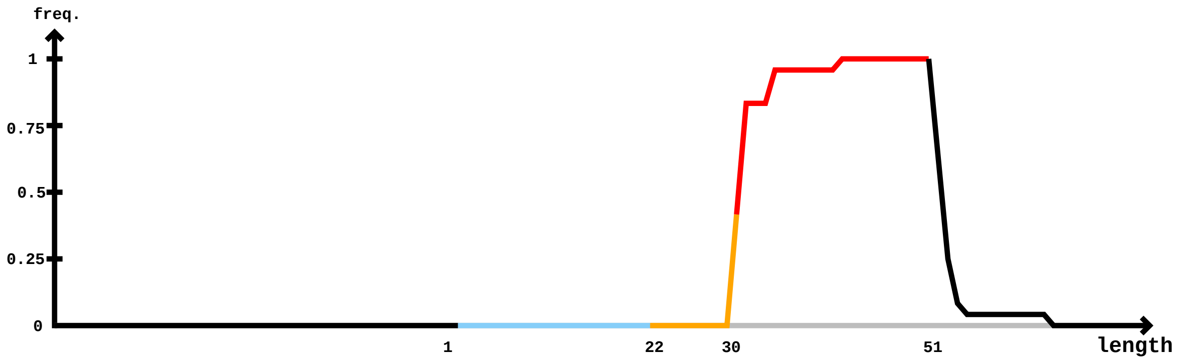

Star Mature

```
5'- ucaacucuucccacacuauuauaucuauugaguuggccaaaaaguuuauucagguuuguuaucuuagggaaagacccaaacgaacuuuuuggccaacccaauauuuuauugu   -3'    exp
    .........................(((((.((((((((((((((((.((..(((((..............)))))..)).)))))))))))))))).))))).........   reads    mm    sample
    .....................................................................aaagacccaaacgaacuuuuu......................   2        0     seq
    .....................................................................aaagacccaaaUgaacuuuuu......................   7        1     seq
    .....................................................................aaagacccaaacgaacuuuuug.....................   1        0     seq
    ......................................................................aagacccaaacgaacuuuuu......................   1        0     seq
    ......................................................................aagacccaaaUgaacuuuuug.....................   3        1     seq
    ......................................................................aagacccaaacgaacuuuuug.....................   3        0     seq
    ......................................................................aagacccaaacgaacuuuuugg....................   3        0     seq
    .........................................................................acccaaacgaacuuuuu......................   1        0     seq
    .........................................................................acccaaacgaacuuuuugU....................   1        1     seq
    .........................................................................acccaaacgaacuuuCuggc...................   1        1     seq
    ................................................................................cgaacuuuuuggccaacccCau..........   1        1     seq
```
